# An Endogenous Staphylococcus aureus CRISPR-Cas System Limits Phage Proliferation and Is Efficiently Excised from the Genome as Part of the SCC*mec* Cassette

**DOI:** 10.1128/spectrum.01277-23

**Published:** 2023-07-05

**Authors:** Kasper Mikkelsen, Janine Zara Bowring, Yong Kai Ng, Frida Svanberg Frisinger, Julie Kjærsgaard Maglegaard, Qiuchun Li, Raphael N. Sieber, Andreas Petersen, Paal Skytt Andersen, Jakob T. Rostøl, Nina Molin Høyland-Kroghsbo, Hanne Ingmer

**Affiliations:** a Department of Veterinary and Animal Sciences, University of Copenhagen, Copenhagen, Denmark; b Department of Bacteria, Parasites and Fungi, Statens Serum Institut, Copenhagen, Denmark; c Jiangsu Key Lab of Zoonosis/Jiangsu Co-Innovation Center for Prevention and Control of Important Animal Infectious Diseases and Zoonoses, Yangzhou University, Yangzhou, China; d Centre for Bacterial Resistance Biology, Imperial College London, London, United Kingdom; e Department of Plant and Environmental Sciences, University of Copenhagen, Frederiksberg, Denmark; Wuhan University

**Keywords:** CRISPR-Cas type III-A, *Staphylococcus aureus*, MRSA, SCC*mec* type V(5C2&5), bacteriophage, CRISPR-Cas, SCC*mec*, type III-A, type V(5C2&5)

## Abstract

CRISPR-Cas is an adaptive immune system that allows bacteria to inactivate mobile genetic elements. Approximately 50% of bacteria harbor CRISPR-Cas; however, in the human pathogen Staphylococcus aureus, CRISPR-Cas loci are less common and often studied in heterologous systems. We analyzed the prevalence of CRISPR-Cas in genomes of methicillin-resistant Staphylococcus aureus (MRSA) strains isolated in Denmark. Only 2.9% of the strains carried CRISPR-Cas systems, but for strains of sequence type ST630, over half were positive. All CRISPR-Cas loci were type III-A and located within the staphylococcal cassette chromosome *mec* (SCC*mec*) type V(5C2&5), conferring β-lactam resistance. Curiously, only 23 different CRISPR spacers were identified in 69 CRISPR-Cas positive strains, and almost identical SCC*mec* cassettes, CRISPR arrays, and *cas* genes are present in staphylococcal species other than S. aureus, suggesting that these were transferred horizontally. For the ST630 strain 110900, we demonstrate that the SCC*mec* cassette containing CRISPR-Cas is excised from the chromosome at high frequency. However, the cassette was not transferable under the conditions investigated. One of the CRISPR spacers targets a late gene in the lytic bacteriophage phiIPLA-RODI, and we show that the system protects against phage infection by reducing phage burst size. However, CRISPR-Cas can be overloaded or circumvented by CRISPR escape mutants. Our results imply that the endogenous type III-A CRISPR-Cas system in S. aureus is active against targeted phages, albeit with low efficacy. This suggests that native S. aureus CRISPR-Cas offers only partial immunity and in nature may work in tandem with other defense systems.

**IMPORTANCE** CRISPR-Cas is an adaptive immune system protecting bacteria and archaea against mobile genetic elements such as phages. In strains of Staphylococcus aureus, CRISPR-Cas is rare, but when present, it is located within the SCC*mec* element, which encodes resistance to methicillin and other β-lactam antibiotics. We show that the element is excisable, suggesting that the CRISPR-Cas locus is transferable. In support of this, we found almost identical CRISPR-Cas-carrying SCC*mec* elements in different species of non-S. aureus staphylococci, indicating that the system is mobile but only rarely acquires new spacers in S. aureus. Additionally, we show that in its endogenous form, the S. aureus CRISPR-Cas is active but inefficient against lytic phages that can overload the system or form escape mutants. Thus, we propose that CRISPR-Cas in S. aureus offers only partial immunity in native systems and so may work with other defense systems to prevent phage-mediated killing.

## INTRODUCTION

Clustered regularly interspaced short palindromic repeats (CRISPR) and CRISPR-associated Cas proteins are microbial adaptive immune systems that protect against invading genetic elements such as phages (1) and plasmids (2). Genetic memory of such prior encounters is stored as spacer sequences in the CRISPR array, and adaptation is accomplished by acquisition of foreign DNA fragments that are inserted as new spacers downstream of the CRISPR leader region (3). The CRISPR array is transcribed as a pre-CRISPR RNA that is processed by Cas proteins into mature CRISPR RNAs (crRNAs). During the interference stage, the crRNAs guide Cas protein complexes to the foreign DNA or RNA sequences by binding matching protospacers, with subsequent invader nucleic acids being destroyed by the Cas nucleases (4).

Despite its ability to protect bacteria from foreign genetic elements, only approximately 40% of all bacteria harbor CRISPR-Cas immune systems (5), and for some bacterial species the system is present in only a subset of strains. One such example is Staphylococcus aureus. It is an opportunistic, human pathogen that naturally colonizes both humans and animals and gives rise to serious, life-threatening infections. Until now, only a few S. aureus strains have been reported to encode CRISPR-Cas systems, and these are almost exclusively positioned within the staphylococcal cassette chromosome *mec* (SCC*mec*) and belong to the type III-A subgroup, which targets both DNA and RNA (6–9). Type III-A CRISPR-Cas activity is dependent on transcription of target sequences and involves specific RNase activity recognizing transcripts of these sequences as well as single-stranded DNase activity, degrading the target DNA (10, 11). In contrast to other CRISPR-Cas systems, type III-A does not rely on a protospacer-adjacent motif (PAM) to distinguish self from nonself. Instead, it depends on mismatches between the protospacer-flanking sequences in the foreign DNA and the CRISPR repeats, where mismatches indicate nonself and allow DNA targeting, whereas extended homology between crRNA and CRISPR DNA indicates self (12). Much of the previous work characterizing the type III-A CRISPR-Cas systems in S. aureus was performed using expression vectors or heterologous expression systems (10, 13, 14), and thus, little is known about the activity of the endogenous S. aureus type III-A CRISPR-Cas systems against invading genetic elements (7).

In S. aureus, strains are subdivided based on multilocus sequence types (STs) and on composition of the *spa* gene, and they are categorized as being either hospital, community, or livestock associated (15, 16). The first CRISPR-Cas element described in S. aureus was reported for a livestock-associated MRSA strain, 08BA02176, with the sequence type ST398 and *spa* type t034 (6). 08BA02176 was isolated in Canada from a human infection and it carried an intact CRISPR-Cas locus with 15 spacers located within the CRISPR array upstream of the *cas* genes and 3 spacers in the downstream array. Interestingly, the CRISPR-Cas locus was located within the SCC*mec* cassette that in MRSA strains carries the *mecA* gene encoding the alternative penicillin-binding protein providing methicillin resistance. In another study, five clinical isolates of MRSA were demonstrated to carry a type III-A CRISPR-Cas system located in the SCC*mec* type V cassette (8). This study showed that type III-A CRISPR-Cas activity depends on transcription and that the Cas proteins Cas10, Csm2, Csm3, Csm4, and Cas6 are required for CRISPR-Cas interference. We recently examined available S. aureus genome sequences for CRISPR-Cas systems and found 35 genomes that encoded a complete type III-A CRISPR-Cas system (7). In all cases, the system was located within the SCC*mec* cassette, and for one strain, we examined the CRISPR-Cas activity and demonstrated CRISPR-Cas-dependent protection against phage infection.

The predominant SCC*mec* cassette that carries the CRISPR-Cas system is of the subtype V(5C2&5) (7), based on the two type 5 *ccr* recombinase genes and the class C2 *mec* gene complex (17, 18). This SCC*mec* has previously shown partial excision *in vivo* via recombination events between the two recombinase alleles (19) and can be partially packaged into ϕ53 phage capsids. However, these phages were unable to transduce methicillin resistance (20).

In addition to S. aureus, CRISPR-Cas systems also appear in other staphylococcal species, including Staphylococcus argenteus, a species recently reclassified from S. aureus strains belonging to ST1850 and ST2250 (8, 21, 22). In the coagulase-negative staphylococci (CoNS), type III-A systems are found in S. epidermidis, S. lugdunensis, S. capitis, and S. warneri (23, 24). Curiously there is extensive homology of *cas* genes and CRISPR spacers between S. aureus and CoNS, leading to the suggestion that there may have been recent exchange of CRISPR-Cas along with SCC*mec* between these species (9, 25).

Here, we report the prevalence of CRISPR-Cas systems in clinical MRSA strains in Denmark and show that type III-A CRISPR-Cas systems are present in more than half of the examined strains belonging to the emerging clone ST630 (26, 27). As in previous studies, the CRISPR-Cas locus is located within the SCC*mec* cassette type V(5C2&5) and has substantial homology to similar elements in the CoNS. Interestingly, we demonstrate that the entire SCC*mec* cassette is excised and circularizes at high frequencies, suggesting that the circular form can be transferred horizontally and that recipient strains acquire CRISPR-Cas phage defense and methicillin resistance simultaneously. However, we did not detect transmission of the SCC*mec* cassette between S. aureus strains using our experimental setup. Further, we show that the endogenous CRISPR-Cas system in the clinical isolate 110900 protects against the lytic phage phiIPLA-RODI, but it can be circumvented either by escape mutations in the phage or by an overload of the system with phage. This implies that the endogenous type III-A CRISPR-Cas systems of S. aureus offer various degrees of protection against phage infections, even when they encode a CRISPR spacer targeting the phage.

## RESULTS

### CRISPR-Cas prevalence in clinical MRSA isolates.

To determine the prevalence of CRISPR-Cas systems in MRSA, we analyzed 1,490 clinical MRSA isolates sequenced at the Danish Statens Serum Institut (SSI). We screened for the presence of the conserved *cas1* and *cas2* genes (28) and typed the CRISPR-Cas systems using the CRISPRCasFinder database (29). Of the 1,490 isolates, 43 (2.9%) contained a complete CRISPR-Cas system, all of which belong to the type III-A system. The most prevalent CRISPR-Cas-positive (CRISPR-Cas^+^) S. aureus clone was ST630, with more than half of the isolates carrying CRISPR-Cas, followed by ST1, ST5, and ST130 (Table 1). As ST630 is an emerging clone in both Denmark and China (26, 27, 30), we included 30 additional ST630 isolates in the analysis. A few isolates were excluded due to inadequate CRISPR array sequencing, bringing the total number of CRISPR-Cas^+^ strains to 69.

**TABLE 1:** CRISPR-Cas prevalence in clinical MRSA isolates

ST	No. of CRISPR-Cas^+^ strains/total	% CRISPR-Cas^+^ strains
ST1	13/183	7.1
ST5	5/132	3.8
ST130	2/83	2.4
ST630	22/40	55
ST3208	1/1	100

Interestingly, for all the S. aureus CRISPR-Cas^+^ clinical isolates, the type III-A CRISPR systems were encoded within SCC*mec* cassettes of the type V(5C2&5) around 3 to 5 kb downstream of *ccrC1* allele 2, within SCC*mec* joining region 1 (J1 region), which is a preferred integration site for plasmids and transposons carrying antibiotic resistance genes (17, 31).

### Spacer patterns of the S. aureus type III-A CRISPR arrays.

**FIG 1:**
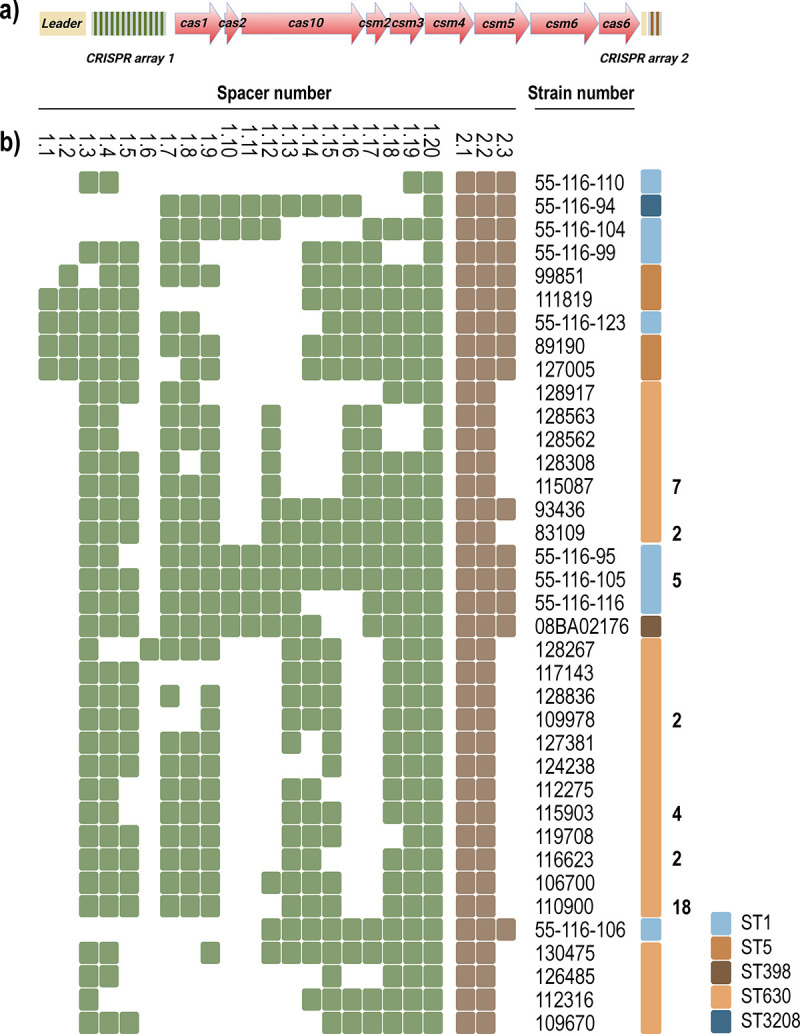
Overview of the type III-A CRISPR-Cas systems examined in S. aureus. (a) Graphic representation of the type III-A CRISPR-Cas system, including leader sequences (yellow), *cas* and *csm* genes (red), and CRISPR array 1 (green/gray) and 2 (brown/gray). (b) Spacer content in CRISPR array 1 and 2 in 69 Danish clinical isolates and 1 reference strain (08BA02176). Green and brown squares symbolize the presence of the spacer in CRISPR array 1 and 2, respectively. STs are designated by color, and the number of isolates that carry identical CRISPR arrays is indicated in bold (the absence of a number indicates 1 strain). The spacers are presented in the order in which they occur in the array.

Among the 69 Danish clinical CRISPR-Cas^+^ isolates and the one reference strain (ST398 isolate 08BA02176), we found 23 distinct spacers present in 37 CRISPR arrays with 20 spacers in CRISPR array 1 (spacers 1.1 to 1.20) and three in CRISPR array 2 (spacers 2.1 to 2.3) (Fig. 1).

The 3′ end of CRISPR arrays is commonly conserved among strains, since these spacers have resided there the longest, until they are eventually lost (32). Accordingly, we found the 3′ end to be highly conserved, with all isolates carrying spacer 1.20 as the final 3′ spacer and 64 of the 70 isolates carrying spacers 1.18 and 1.19 immediately upstream (Fig. 1). In the 5′ region, 62 of 70 isolates had spacer 1.3 as the 5′ spacer, and this was succeeded by spacer 1.4 in 60 of these arrays, whereas five of our isolates had spacers 1.1 and 1.2 as 5′ spacers (Fig. 1b). The remaining three isolates had middle-array spacers from other isolates as their 5′ spacers. In active CRISPR-Cas systems, spacer acquisition commonly occurs at the 5′ end of the CRISPR array, resulting in the highest diversity at this end of the array compared to CRISPR arrays with a recent common origin (33, 34). Surprisingly, we observed the highest diversity in the center of the array, ranging from isolate 55-116-110, which holds only 5′- and 3′-end spacers of other strains in its CRISPR 1 array, to others (e.g., 55-116-105) containing nearly exclusively middle-array spacers. Thus, our results suggest that there is only limited incorporation of new spacers in the CRISPR arrays and that adaptation may involve acquisition of spacers in the middle of the array.

Next, we compared CRISPR spacer composition with ST clonality. We found that CRISPR arrays in ST630 isolates have the 1.3 spacer at the 5′ end, while ST5 arrays start with either the 1.1 or 1.2 spacer. Additionally, the 1.10 and 1.11 spacers are limited to ST1, ST3208, and ST398 isolates. Similarly, for CRISPR array 2, all ST630 isolates but one carry only the 2.1 and 2.2 spacers and all non-ST630 strains carry an additional 2.3 spacer. These observations along with the lack of diversity in the 5′ end of CRISPR array 1, and the presence of only 23 different spacers in the two CRISPR arrays shared between 70 strains, suggest that the arrays have a common origin via horizontal transfer and that adaptation of new spacers is infrequent and can occur at noncanonical sites, e.g., in the middle of the array in these S. aureus strains.

### Spacers found in Danish MRSA isolates target staphylococcal phages and plasmids.

To investigate the target protospacers of the CRISPR-Cas systems in Danish MRSA isolates, we searched the NCBI database for spacer homology. Here, we found that 7 of the 23 spacers had homology to known mobile genetic elements (Table 2), where spacers 1.9, 1.14, 1.18, and 2.2 target phages and 1.5, 1.19, and 1.20 target plasmids. Importantly, of these 7 protospacers, 6 are located within annotated open reading frames and are complementary to the respective mRNA transcripts. This reflects the fact that type III-A CRISPR-Cas systems target transcripts of invading mobile elements (8, 35). Spacer 1.20 covers a bidirectional promoter region but might be functional only during transcription of the *parA* gene due to the aforementioned strand bias.

**TABLE 2:** Spacer targets^*a*^

Spacer	Description	Identity (bp match)	Target	Accession no.	Comments
1.5	Clostridium botulinum Prevot_594 plasmid pCBH	30/35	Hypothetical protein	CP006901.1	Homologous to template strand

1.9^*b*^	Staphylococcus phage phiIPLA-RODI	33/35	Hypothetical protein	KP027446.1	Homologous to template strand
	Staphylococcus phage Stab20	33/35		LR215718.1	
	Staphylococcus phage K	32/35		KF766114.1	

1.14	Staphylococcus phage phiIPLA-C1C	33/36	TreK hypothetical protein	KP027447.1	Homologous to template strand
	Staphylococcus phage phiIBB-SEP1	33/36		NC_041928.1	
	Staphylococcus virus BESEP5	33/36		MT596502.1	

1.18^*b*^	Staphylococcus phage GRCS	33/36	DNA encapsidation/packaging protein	NC_023550.1	Homologous to template strand
	Staphylococcus phage BP39	32/36		KM366100.1	

1.19	Staphylococcus sp. strain CDC3 plasmid SAP020A	32/34	Hypothetical protein	GQ900386.1	Homologous to template strand

1.20	Staphylococcus haemolyticus strain NY5 plasmid pNY5A	36/36	Bidirectional promoter region of ParA family protein and replication initiator protein RepA	CP078160.1	Protospacer is located within a bidirectional promoter region
	Staphylococcus epidermidis strain NCCP 16828 plasmid	30/36		CP043848.1	
	Staphylococcus epidermidis plasmid pHD75-1_1	29/36		CP052942.1	

2.2	Uncultured *Caudovirales* phage clone 7F_7	33/37	DNA-directed RNA polymerase subunit alpha	MF417907.1	Homologous to template strand
	Staphylococcus phage IME1365_01	33/37		KY653129.1	
	Uncultured *Caudovirales* phage clone 7F_13	32/37		MF417958.1	

a Hits from a BLASTn search of spacers 1.1 to 2.3 from CRISPR arrays 1 and 2 against the NCBI database are shown, with annotated identity and protein and strand targets.

b Only hits matching known mobile genetic elements are shown.

The spacer ranges in length from 32 to 39 bp and, in all except one case, carries two or more mismatches to the identified protospacer sequence (Table 2).

### Type III-A CRISPR-Cas is located in the SCC*mec* cassette and is present in various staphylococcal species.

**FIG 2:**
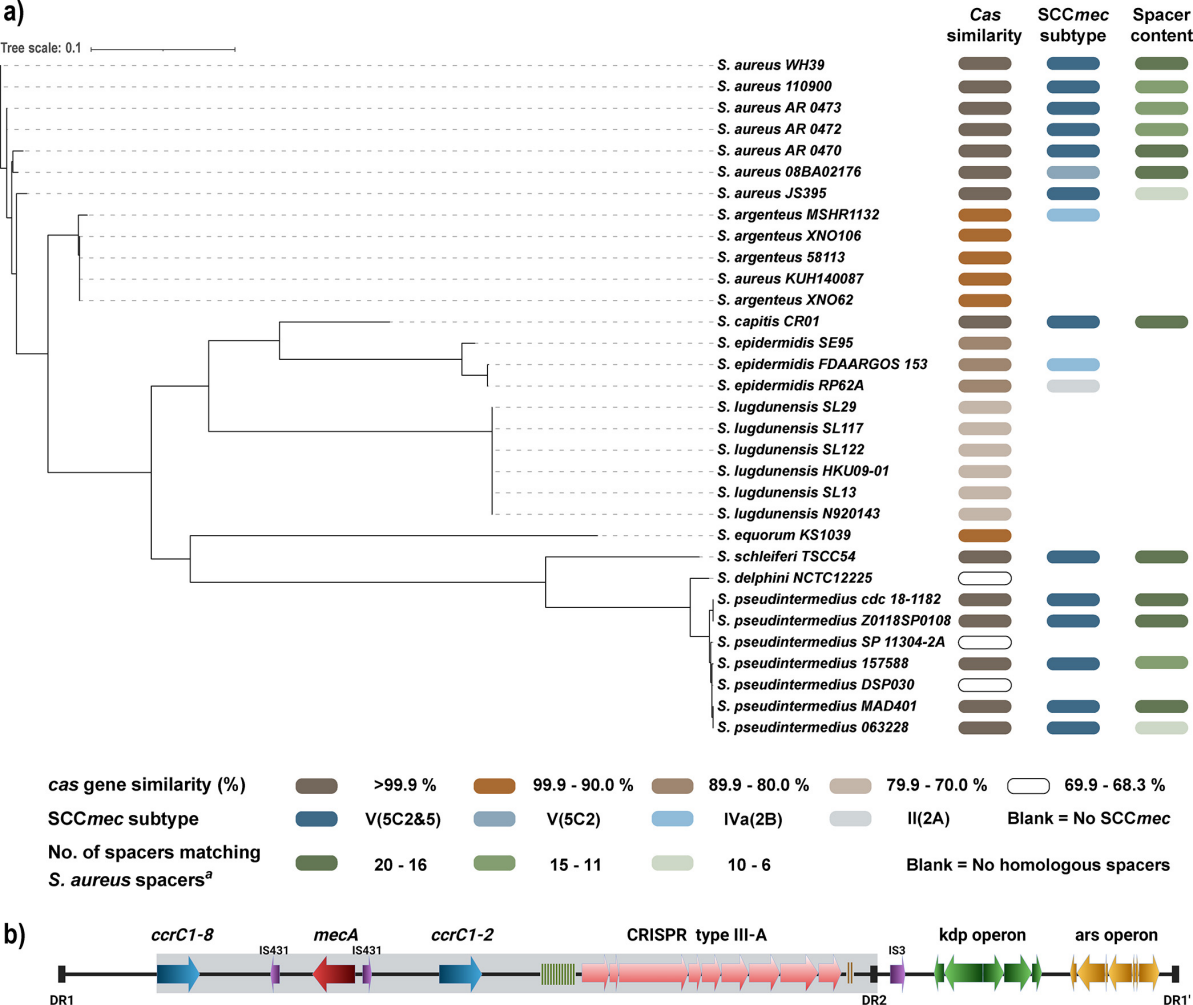
The SCC*mec* type V(5C2&5), harboring type III-A CRISPR-Cas, is shared between staphylococcal species. (a) Phylogenetic core genome SNP tree based on hits from a BLASTn search of the *cas* gene sequence (*cas1* to *cas6*) of strain 110900. *cas* gene percent identity, SCC subtype, and numbers of S. aureus spacers are indicated at the bottom. a, sum of spacers homologous to the S. aureus spacers (1.1 to 2.3) found in staphylococcal isolates. (b) Graphic presentation of SCCmec type V(5C2&5) with the highly conserved region (gray shading).

To further explore type III-A CRISPR-Cas homology within staphylococci, we carried out BLAST analysis of the sequence covering *cas1-6* of the S. aureus strain 110900 (27) against the NCBI database. Based on the hits, we constructed a phylogenetic tree and compared them based on their core genomes (Fig. 2a). Also, we assessed their *cas* gene identity (percent identity compared to the *cas1-6* sequence of strain 110900), SCC*mec* subtypes, and whether they carried any homologues of the 23 S. aureus spacers identified in our screen (1.1 to 2.3). We found that S. aureus strains share almost identical *cas* gene sequences, with >99.9% identity at the nucleotide level. Interestingly, other staphylococcal species as diverse as S. capitis, S. schleiferi, and S. pseudintermedius likewise contain *cas* gene sequences that are nearly identical (>99.9%) to that of S. aureus strain 110900. Additionally, strains of *S. argenteus* and S. equorum share 94% identity to the S. aureus *cas1-6* gene sequence followed by the S. epidermidis (89%) and *S. lugdunensis* (76%) strains (Fig. 2a, *cas* similarity column).

In addition to the CRISPR-Cas locus, strains of *S. capitis*, *S. schleiferi*, and S. pseudintermedius carry SCC*mec* type V(5C2&5) cassettes nearly identical to that of strain 110900 (Fig. 2b). They share 99% identity in the 32-kb region from the *ccrC1-8* to direct repeat 2 (DR2), which includes both recombinase genes, *mecA*, and the entire CRISPR-Cas system (Fig. 2b, gray-shaded area). Also, we found that these strains carry spacers that are highly similar to those found in the S. aureus strains screened earlier in this study (>16), including all of the spacers identified in S. aureus strain 110900. To sum up, the high degree of identity between S. aureus 110900 SCC*mec* type V(5C2&5) (including *cas* genes and CRISPR arrays) and SCC*mec* type V(5C2&5) of other staphylococcal species strongly points to incidences of intra- and interspecies horizontal transfer of the SCC*mec* cassette together with the CRISPR-Cas system.

Based on the core genome sequences, S. aureus and S. pseudintermedius are most distantly related (Fig. 2a). Interestingly, S. pseudintermedius share type III-A *cas* genes with either high (>99.9%) or low (<70%) identity to the S. aureus strain 110900 coinciding with the CRISPR system being placed inside the SCC*mec* or elsewhere on the S. pseudintermedius chromosome, respectively. This further supports the idea that the type III-A CRISPR-Cas system found in S. aureus is transferable between staphylococcal species and likely is mobilized via the SCC*mec* type V(5C2&5).

### Circularization of the SCC*mec* type V(5C2&5) cassette carrying the CRISPR-Cas locus.

**FIG 3:**
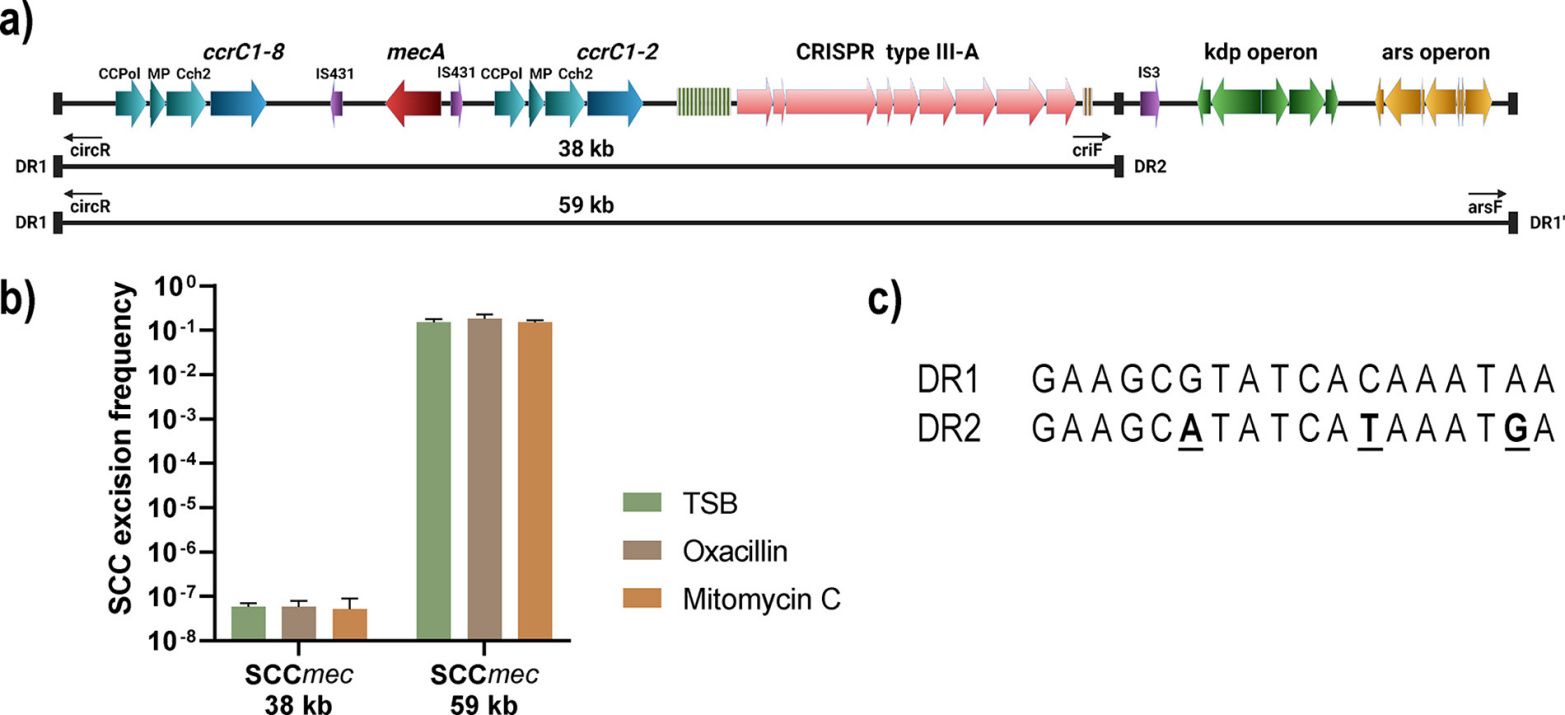
Excision of SCCmec. (a) The SCC*mec* type V(5C2&5) of *S. aureus* strain 1109000 with predicted circularizable fragments. The primers and location of DRs are shown below the SCC*mec*. (b) Excision frequencies of the 38- and 59-kb SCC*mec* fragments compared to chromosomal DNA quantities (*n* = 3). The primer pairs criF/circR and arsF/circR were used for detecting the 38-kb and 59-kb fragments, respectively, as shown in panel a. (c) Alignment of DR1 and DR2, with mismatches highlighted in bold and underlined in the DR2 sequence.

SCC*mec* cassettes in S. aureus have previously been shown to be excised from the genome (36), and therefore, we examined if the SCC*mec* cassette containing the CRISPR-Cas locus was excisable in strain 110900. The SCC*mec* is flanked by an upstream DR and two downstream DRs (DR2 and DR1′) (Fig. 3a) that can potentially recombine to form extrachromosomal circles (37). If so, two distinct circularized fragments may form, both of which contain the CRISPR-Cas system along with the *mecA* gene, namely, a 38-kb fragment arising via recombination of DR1/DR2 or a 59-kb fragment via recombination of the DR1/DR1′ pair (Fig. 3a). We designed primers to span the junction of the two putative circular SCC*mec* entities and used qPCR to quantify levels of excised, circularized SCC*mec* normalized to the amount of the *adsA* gene, located 1 kb upstream of SCC*mec*. The 59-kb fragment generated by excision of the entire SCC*mec* cassette involving DR1/DR1′ recombination showed a relatively high excision and circularization frequency of 10^−1^ relative to the abundance of the chromosomal control (Fig. 3b). Next we hypothesized that specific environmental signals such as antibiotics may trigger or enhance excision, as previously observed for the SCC*mec* type V(5C2&5) in Staphylococcus haemolyticus (37). However, circularization frequency was not affected by subinhibitory concentrations of the β-lactam antibiotic oxacillin or by the DNA-damaging agent mitomycin C. The excision frequency of the 38-kb fragment was approximately 10^−7^ with or without antibiotics, making it 6 orders of magnitude less frequent than excision of the entire cassette. This difference is probably due to the three-nucleotide mismatch between DR1 and DR2 (Fig. 3c).

In S. aureus, horizontal gene transfer is often facilitated by phages (38), and we therefore tested if the circularized SCC*mec* could be transferred by phage transduction. To this end, we infected strain 110900 with the transducing phage ϕ11. We used the resulting phage lysates, containing the progeny phages, to infect S. aureus strains RN4220, 8325-4 ϕ11, and Newman and selected for transductants that had acquired the *mecA* gene and thereby would become oxacillin resistant. Despite repeated attempts, we did not detect any transductants. Thus, under our experimental conditions, the SCC*mec* cassette is not transduced via phage ϕ11.

### The ST630 type III-A CRISPR-Cas system is active but inefficient against phage infection.

**FIG 4:**
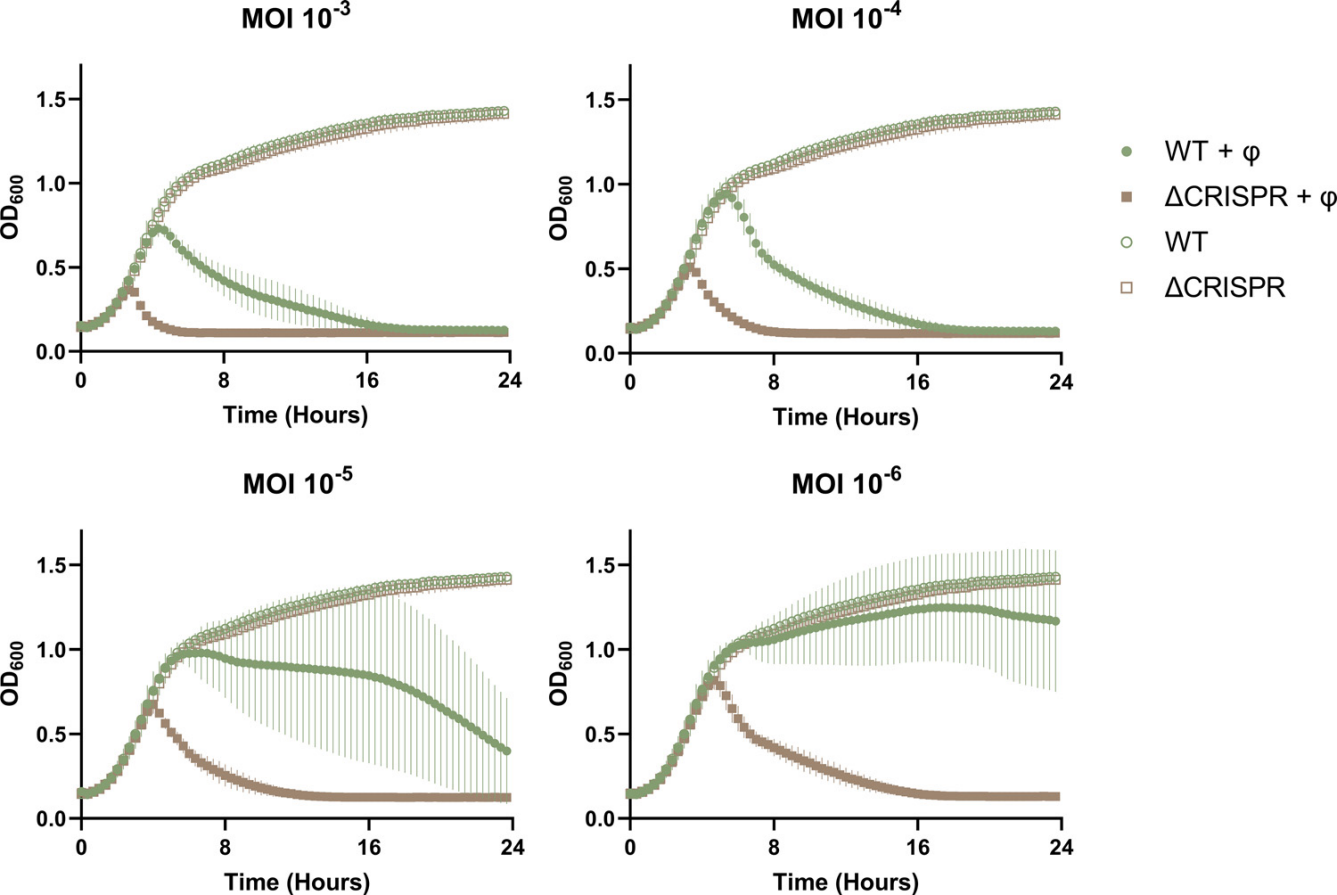
CRISPR-Cas activity against phage phiIPLA-RODI during liquid infection. Cultures (OD_600_, 0.15) of 110900 WT (green) or the ΔCRISPR mutant (brown) were grown with (closed symbols) or without (open symbols) increasing MOIs of phiIPLA-RODI phage in 1:1 TSB-phage buffer, with OD_600_ being monitored every 20 min for 24 h (*n* = 3; 5 technical replicates).

To examine the anti-phage activity provided by the S. aureus type III-A CRISPR-Cas system, we deleted the CRISPR-Cas locus in strain 110900 and infected wild-type (WT) and ΔCRISPR mutant cells with the lytic phage phiIPLA-RODI, which is targeted by spacer 1.9 in the CRISPR array (Table 2). At a multiplicity of infection (MOI) of 10^−6^ (1 phage to 10^6^ bacteria), strain 110900 survived the infection, whereas the ΔCRISPR mutant failed to grow (Fig. 4). At higher initial phage concentrations (MOI of 10^−3^ and 10^−4^), the overall killing of the ΔCRISPR mutant correspondingly happened earlier than for the WT strain. Interestingly, for the WT 110900, there was a large variation between the technical replicates when cells were infected at low MOI of 10^−5^ and 10^−6^. This variation was a consequence of some bacterial cultures lysing from the phage infection, while others survived for longer periods of time. Thus, at low MOI, the CRISPR-Cas system protects 110900 against phage killing, but at higher MOI, the system is overwhelmed.

As we had observed high variability between technical replicate cultures in the CRISPR-Cas-mediated protection against phages at low MOI, we investigated this further. Thus, we repeated the experiment whose results are shown in Fig. 4 by infecting strain 110900 with phiIPLA-RODI at an MOI of 10^−6^, and at the end of the experiment (24 h), we sequenced the phages in the wells of the five technical replicates where lysis had occurred. In three of the replicates, we found that the phages had an identical 476-bp deletion covering AVU41_gp213, AVU41_gp212, and the intergenic region upstream of these two genes (see Fig. S1 in the supplemental material). Importantly, AVU41_gp213 is the phiIPLA-RODI gene that is targeted by the 110900 CRISPR spacer 1.9. Thus, escape mutants of phiIPLA-RODI can circumvent the CRISPR-Cas activity.

**FIG 5:**
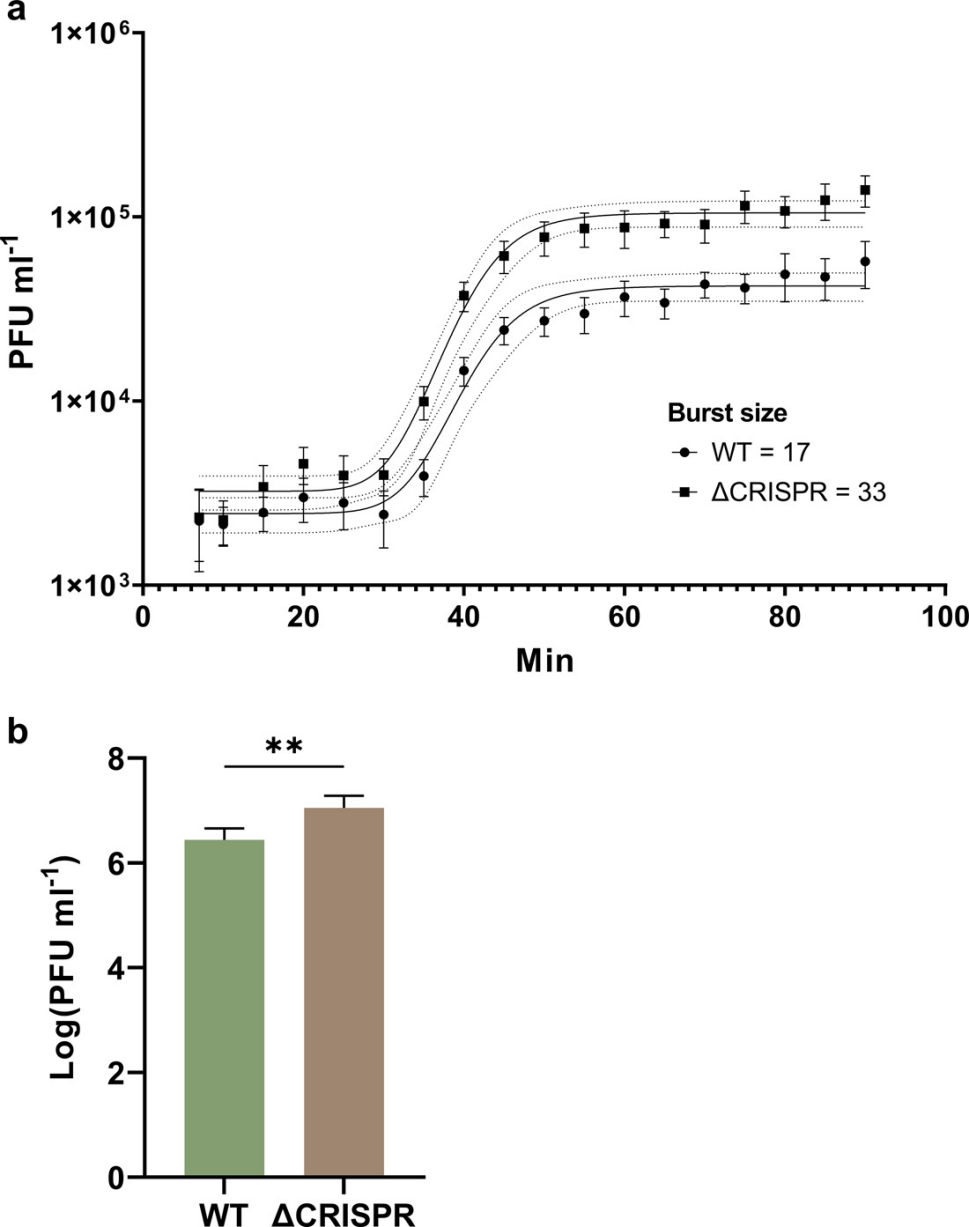
CRISPR-Cas reduces phiIPLA-RODI burst size. (a) One-step growth curves of phiIPLA-RODI using WT 110900 or the ΔCRISPR mutant as a propagative strain determined from 5 biological replicates (standard errors of the means [SEM] are shown) and compared with a paired two-tailed *t* test (****, *P* < 0.0001). The nonlinear regression sigmoidal curves were fitted using GraphPad Prism with least-squares regression. Burst size was calculated as the top plateau average divided by the bottom plateau average. (b) Plaque formation of phiIPLA-RODI using either WT 110900 or the ΔCRISPR mutant as the recipient. Data are mean log transformed PFU per milliliter for 4 biological replicates, with SD. **, *P* = 0.0087 (unpaired *t* test). The EOP of phiIPLA-RODI on 110900 WT was 29% of that on the ΔCRISPR mutant.

We also examined the burst size of phiIPLA-RODI when infecting either WT 110900 or the ΔCRISPR mutant, using a one-step growth curve (Fig. 5a). We found that the burst size of the phage was 17 when the WT was infected but 33 in the ΔCRISPR mutant, showing that CRISPR-Cas reduces the average amount of phage progeny produced. Similarly, in a plaque assay when the two strains were used as recipients for a phage titer determination, significantly more PFU were detected on the ΔCRISPR mutant than the WT, equating to an efficiency of plaquing (EOP) of 29% on the WT recipient (Fig. 5b). Thus, our data show that the S. aureus strain 110900 CRISPR-Cas type III-A system is active and offers protection against low-MOI infection; however, the system can be overwhelmed and can select for phage escape mutants where the targeted region has been deleted.

## DISCUSSION

In S. aureus, CRISPR-Cas systems are found in only a subset of strains. Here, we show that in Danish clinical MRSA isolates, 2.9% were CRISPR-Cas positive, with all isolates carrying the type III-A system. This resembles the prevalence of CRISPR-Cas in S. aureus in previous studies. In the work of Cao et al. (8), the prevalence was found to be 0.94%, and in a previous screen looking for CRISPR-Cas among 12,582 S. aureus sequences in the NCBI database subjected to whole-genome sequencing, we found 35 sequences that carry CRISPR-Cas, all of which are type III-A and 30 of which are located in SCC*mec* type V (7). This suggests a strong bias for the CRISPR-Cas locus to be located in SCC*mec* type V and thereby be more prevalent in MRSA isolates (2.9%) than in S. aureus in general (35/12,582 [0.3%]). The MRSA clone with the highest CRISPR-Cas frequency was the emerging ST630 clone, where more than 50% of the isolates were CRISPR-Cas^+^. Inspection of the ST630 CRISPR arrays revealed conserved spacers at the 5′ end of the array, and curiously, CRISPR array 1 mainly differed at the center of the array. This supports recent findings that the order of spacers in CRISPR arrays may arise from a combination of events, including middle-array insertion, recombination within or between arrays, and horizontal transfer of all or part of the array (39). As spacer adaptation via Cas1 and Cas2 in Staphylococcus has been observed only with overexpression in an inducible setting (40), the type III-A system could rely on alternative mechanisms to adapt new spacers, such as recombination between CRISPR spacers and their cognate protospacer (41) or integration and excision of temperate phages (42). Also, low CRISPR adaptation frequencies in S. aureus may prevent self-targeting and may permit horizontal acquisition of useful genes, such as phage-inducible chromosomal islands (PICIs) or plasmids. Indeed, S. aureus strains carry many prophages and PICIs on their genomes that are important for virulence (43, 44).

We further observed that there is a high degree of conservation of spacers across staphylococcal species (Fig. 2a) and between S. aureus strains (Fig. 1). This has previously been observed for S. aureus strains (7) as well as between staphylococcal species (8, 45). Besides suggesting a low frequency of adaptation events of the type III-A CRISPR-Cas system, the interspecies conservation of spacers as well as *cas* genes indicates horizontal transfer between staphylococci. As the type III-A CRISPR-Cas locus is located within similar SCC*mec* type V(5C2&5) cassettes across species, including *S. capitis*, *S. schleiferi*, and S. pseudintermedius, this supports the notion that the SCC*mec* cassette is of non-S. aureus origin (46). Indeed, we observed that the S. pseudintermedius SCC*mec* containing CRISPR-Cas has an identity of 99% to that of S. aureus strain 110900. The occurrence of highly conserved SCC*mec* elements containing CRISPR-Cas in strains of sequence type ST630 could be related to their unusual composition of cell wall teichoic acids, which has been proposed to enable horizontal gene transfer between coagulase-negative staphylococci and S. aureus (26).

CRISPR-Cas systems have previously been associated with mobile genetic elements, including plasmids, genomic islands, and transposons (47, 48). In S. aureus, the SCC*mec* cassette has been reported to be excised from the genome (36), and we also observed that the entire SCC*mec* type V(5C2&5) including CRISPR-Cas was excised at a high frequency. Generally, it is unknown how SCC*mec* cassettes are transferred, but proposed routes include conjugative plasmids, transduction at low frequencies, and, most recently, natural transformation (49). We attempted to transduce the SCC*mec* type V(5C2&5) with the general transducing phage ϕ11 and transfer the element via natural competence, but in both cases, we were unable to detect transfer. This is likely caused by the limited packaging capacity of ϕ11, which may be unable to accommodate the 59-kb SCC*mec*. Furthermore, since the 38-kb fragment rarely circularizes, any phage-mediated transfer of this smaller fragment would probably be below the detection limit. A recent study identified S. aureus transfer of an SCC*mec* cassette by natural transformation (50); however, we were unable to show natural transformation of the SCC*mec* and CRISPR-Cas in our strains. Thus, it is unclear whether and how SCC*mec* cassettes carrying CRISPR-Cas are transferred between S. aureus strains.

Employing one of the strains identified in the screen, we analyzed the activity of the endogenous CRISPR-Cas system of the ST630 strain 110900 against phage phiIPLA-RODI, which is targeted by spacer 1.9 (Fig. 1 and Table 2). The one-step growth curves of phiIPLA-RODI confirmed that CRISPR-Cas confers some protection against phage proliferation, with phiIPLA-RODI having an approximately 2-fold-greater burst size than the WT strain. A previous study also indicated that type III-A CRISPR-Cas systems may influence burst size, with the temperate phage ϕNM1γ6 yielding a burst size of ~5 PFU when propagated in a strain encoding a targeting spacer compared to ~85 PFU in the absence of targeting spacers (10). Whereas the latter experiments were performed in S. aureus using a plasmid-encoded S. epidermidis CRISPR-Cas system with an engineered spacer targeting a virulent version of temperate S. aureus phage ϕNM1, our results show that native type III-A CRISPR-Cas systems also reduce average phage burst size.

In general, we found that the WT 110900 strain was more resistant to phage infection than the ΔCRISPR mutant (Fig. 4 and 5b, respectively). However, the CRISPR-mediated protection was greatly dependent on MOI, where the system was overwhelmed at MOI of 10^−4^ and higher. At lower MOI, there was great variation in CRISPR-Cas-mediated protection between replicates, with some cultures surviving while others were killed by phage escape mutants that carried deletions of the 35-bp 1.9 protospacer sequence. This could be linked to the fact that spacer 1.9 targets a gene in the long terminal repeats of phiIPLA-RODI, which is presumably a late-expressed gene. For late-expressed genes, it has been shown that both the DNase and RNases of the type III-A CRISPR-Cas system are required to prevent phage-mediated killing and that many phage genomes can accumulate before targeted degradation occurs (10). This could increase the likelihood of escape mutants occurring during phage replication. Escape from the type III-A system has been shown to stem from large deletions of the invading mobile genetic elements that include the protospacer region (51). It is curious that we saw identical regions deleted in 3 of our 5 phage escape mutants, and initially we thought that this deletion mutant of phiIPLA-RODI was present in the ancestral phage pool. However, we did not observe the escape mutations in our phiIPLA-RODI stock, and indeed, with our experimental setup, the likelihood of the mutations preexisting in our phage inoculum is low, given that the number of phages added was approximately 7 PFU with the MOI of 10^−6^. An additional explanation for the conservation of the deletion mutants could be that this region is a hot spot for recombination. Despite incomplete CRISPR-Cas-mediated phage defense, the emergence of phage escape mutants demonstrates that the native CRISPR-Cas system does confer an evolutionary restraint for phages.

Interestingly, the level of protection provided by the endogenous CRISPR-Cas system was much less than reported in the studies in which heterologous expression vectors were used. In a recent study of the S. epidermidis type III-A CRISPR system expressed from a plasmid in an S. aureus strain, there was a 10^3^- to 10^4^-PFU mL^−1^ reduction in phage abundance compared to that in the WT S. aureus strain without the plasmid (14). Here, we found a CRISPR-dependent reduction of ~10^1^ PFU mL^−1^, implying that this endogenous S. aureus CRISPR-Cas confers less protection. However, this is also lower than in a previous study in which we examined the type III-A CRISPR-Cas system encoded by S. aureus ST630 strain TZ0912 (7). Differences in CRISPR-Cas expression, spacer placement, and composition could underlie some of the difference in CRISPR-Cas targeting efficiency; the TZ0912 spacer 6 has 34/35 bp identical to the phiIPLA-RODI target gene, while the 110900 spacer 1.9 has 33/35.

Collectively, we found that the endogenous CRISPR-Cas type III-A system in S. aureus is active in protecting against phages but does so inefficiently. Furthermore, the conservation of spacers between S. aureus strains and even between staphylococcal species indicates that acquisition of new spacers is a rare event. When it does happen, however, it may involve recombination between invading mobile genetic elements and the excised copies of the SCC*mec* type V(5C2&5) (41). The partial protection offered by the endogenous CRISPR-Cas type III-A system in S. aureus may not be surprising and may be just one element of a multilayered protection system. Indeed, the bioinformatic tools DefenseFinder and PADLOC show that the 110900 strain carries additional defense systems, including restriction modification, abortive infection, serine/threonine kinase, and Dodola systems (Table S1) (52–55). Therefore, these systems are likely to defend against phage killing in a synergistic or additive manner not observed in heterologous systems (56). Likewise, CRISPR-Cas may be differentially regulated depending on environmental conditions; e.g., activity could be enhanced in biofilms, where bacteria may encounter phages in low numbers at which the CRISPR-Cas system exerts effective protection. Notably, in Pseudomonas aeruginosa, the activity of the type I-F CRISPR-Cas system is enhanced by a reduced bacterial growth rate (57), and as bacteria within biofilms grow slowly, they may have increased CRISPR-Cas activity. Overall, our findings indicate that the native S. aureus type III-A CRISPR-Cas system provides partial phage immunity and is likely part of the wider phage defense arsenal working in synergy. Moreover, our finding that the entire type V(5C2&5) SCC*mec*, including CRISPR-Cas, was excised at a high frequency suggests that methicillin resistance and CRISPR-Cas-mediated phage defense systems may be mobilized simultaneously under yet-unrecognized conditions. This could prove a challenge for efficient use of phages for therapy of MRSA infections, particularly infections caused by the emerging clone ST630, in which we found 50% of the isolates to harbor CRISPR-Cas.

## MATERIALS AND METHODS

### Isolate collection and classification.

All bacterial isolates were submitted to Statens Serum Institut as part of the national surveillance program for MRSA in Denmark between 27 October 2017 and 12 March 2019. They were subjected to whole-genome sequencing and analyzed as described below. All isolates were typed at Statens Serum Institut as part of the national MRSA surveillance program. The initial screen included 1,504 isolates; however, as one isolate proved to be S. epidermidis and ST1850 and ST2250 were recently reannotated as *S. argenteus* (22), these were excluded from analysis. Thus, the number of MRSA isolates included in the study was reduced to 1,490. Isolates that contain *cas1* and *cas2* genes were selected for further exploration. The genome sequence of strain 110900 has been published (27).

### Whole-genome sequencing and analysis.

All isolates were subjected to whole-genome sequencing on an Illumina MiSeq platform with 2 × 251-bp paired-end reads. Isolates were assembled using SKESA or SPAdes. CRISPRCasFinder was used to identify isolates that contain the *cas1* and *cas2* genes (29).

### Spacer analysis.

Spacers from CRISPR-Cas^+^ isolates were aligned using Clustal Omega and clustered by hierarchical clustering by Ward’s method based on a 2-indel difference calculated by ape version 5.0. A maximum-likelihood phylogenetic tree was calculated using IQ-TREE with default settings running with bootstrap of 100 from single nucleotide polymorphisms (SNPs) called and filtered by NASP version 1.0. A dendrogram of 37 strains was constructed using the unweighted pair group method with arithmetic means (UPGMA). Visualization of spacers was performed using plotly Graph Objects (58) and adjusted in Adobe Illustrator. For spacer sequences, see Table S1.

### Identification of spacer targets.

The spacer groups were subjected to a BLAST search against the entire NCBI database using the BLASTn algorithm on 7 January 2021. Bacterial isolates carrying the CRISPR-Cas system and CRISPR arrays were not classified as protospacers. Hits representative of the targeted protospacer are included in Table 1.

### Chromosomal deletion of the CRISPR system.

Sequences of ~650 bp upstream and downstream of the CRISPR locus in strain 110900 were PCR amplified from purified 110900 chromosomal DNA with overlapping sequences: upstream, pIMAY-Z_CRISPR_uF/pIMAY-Z_CRISPR_uR; downstream, pIMAY-Z_CRISPR_dF/pIMAY-Z_CRISPR_dR (Table S2). These fragments were used in a second overlap extension PCR with primers pIMAY-Z_CRISPR_uF and pIMAY-Z_CRISPR_dR to construct an ~1.3-kb fragment, which was cloned into the PCR-amplified pIMAY-Z plasmid backbone using a homemade seamless ligation cloning extract (SLiCE) as described by Monk and Stinear (59). The mutant was created by following the step-by-step protocol also provided by Monk and Stinear (59).

### Liquid infection assay.

Strain 110900 and the ΔCRISPR mutant were grown in tryptic soy broth (TSB) overnight and diluted to an optical density at 600 nm (OD_600_) of 0.15. The strains were then transferred into honeycomb bioscreen plates (95025BIO) in 125-μL aliquots, and 125 μL of various phage phiIPLA-RODI lysate dilutions in phage buffer (1 mM MgSO_4_, 4 mM CaCl_2_, 50 mM Tris-HCl [pH 8], 0.1 M NaCl), equivalent to an MOI of 1 to 10^−7^, was added. The OD_600_ of each well was measured in a Bioscreen C instrument (Oy Growth Curves Ab Ltd.), with measurements every 20 min for 24 h, at 30°C with shaking. Shaking was paused for 5 s before each reading. For each experiment, 5 technical replicates of each condition were included and 3 biological replicates were performed in total. Positive bacterial growth controls consisting of bacterial culture and phage buffer without phage (125 μL:125 μL) and negative controls consisting of TSB and phage buffer (125 μL:125 μL) were included in each run. Results were plotted on an *xy* graph (GraphPad Prism 9) as means and standard deviations (SD).

### Phage genome sequencing.

DNA was extracted from a sample of the original WT phiIPLA-RODI phage lysate and 5 different phiIPLA-RODI samples grown on the CRISPR-Cas-positive 110900 strain for 24 h in the bioscreen experiments, using the GenElute bacterial genomic DNA extraction kit (Sigma-Aldrich), using the standard protocol with 200 μL phage lysate taken directly from the bioscreen plates. All isolates were subjected to whole-genome sequencing on an Illumina MiSeq platform with 2 × 251-bp paired-end reads. Sequences were assembled to the reference phiIPLA-RODI genome (NC_028765) using Geneious Prime with the BBDUK trimmer plug-in. SNPs and variations were called using Geneious Prime, excluding regions of high or low coverage, with any SNPs/variations present in the WT sample being excluded from further analysis.

### SCC*mec* excision frequency assay.

110900 was grown overnight and diluted to an OD_600_ of 0.05. Cultures were grown for 2 h at 37°C, at which point the cultures were either treated with antibiotics (oxacillin or mitomycin C, 0.5 μg/mL) or left untreated. After 1 h additional incubation at 37°C, 1 mL of culture was withdrawn, and chromosomal DNA was extracted using the DNeasy blood and tissue kit (Qiagen). The samples were normalized according to DNA concentration and diluted 1:5 before being used in qPCRs. qPCRs were set up using the FastStart Essential DNA Green master kit (Roche), using three different primer pairs: criF/circR (38-kb fragment circularization), arsF/circR (59-kb fragment circularization), and adsAF/adsAR (chromosomal reference). For primer sequences, see Table S2. Reactions were run on a LightCycler 96 instrument (Roche), and data were analyzed using the 2^–ΔΔ^*^CT^* method (60). The PCR product sequences were confirmed by cloning the PCR products into the pCR4Blunt-TOPO vector by TOPO cloning (Thermo Fisher) and sequenced using the M13 reverse primer site by Sanger sequencing (Eurofins).

### SCC*mec* transduction experiment.

ST630 isolate 110900 was grown overnight and diluted to an OD_600_ of 0.05. For initial phage infection of strain 110900, 200-mL cultures were grown at 37°C to an OD_600_ of 0.15, and the cells were collected, before resuspension in 1:1 TSB-phage buffer at a final volume of 100 mL. The cultures were infected with ϕ11 at various MOI at 30°C for 4 h. If visible lysis of the culture was not complete, the culture was further incubated at room temperature overnight. Once visible lysis was complete, the lysates were filtered to remove any remaining bacterial cells (0.22-μm filters; Millipore Stericup; with polyethersulfone (PES) membrane).

Lysates were precipitated to increase the concentration of phage particles and any potential CRISPR/SCC*mec* transductant particles. Lysates were incubated with DNase (2.5 U mL^−1^) and RNase (1 μg mL^−1^) at 37°C for 1 h. NaCl was added (58.4 g L^−1^) and lysates incubated on ice with shaking for 1 h. Lysates were centrifuged at 11,000 × *g* for 10 min at 4°C and the supernatant collected. Polyethylene glycol 8000 (PEG 8000) was added at 10% (wt/vol), and the lysates were incubated on ice at 4°C overnight. The lysates were centrifuged at 11,000 × *g* for 10 min at 4°C, and the supernatant was discarded. Phage precipitants were resuspended in 1.6 mL phage buffer and quantified by titration methods.

Transduction assays were attempted using recipient strains RN4220, 8325-4 ϕ11, and Newman. Briefly, overnight cultures of recipients were diluted to an OD_600_ of 0.05 and grown to an OD_600_ of 1.4, before 1 mL of recipient, 100 μL of phage lysate and 4.4 mM CaCl_2_ were incubated at 37°C for 20 min. The mixture was plated in 3 mL tryptic soy agar (TSA) top agar (50% agar) on TSA with oxacillin (0.4 μg mL^−1^) and sodium citrate (17 mM). Plates were incubated at 37°C for 24 h and checked for colonies; if no colonies were observed, the plates were incubated for an additional 24 h and checked again.

### One-step growth curves.

One-step growth curves were performed essentially as previously described (61). Briefly, bacterial propagative strains were subcultured and grown to mid-log phase before addition of 5 × 10^5^ PFU mL^−1^ phiIPLA-RODI to 9.9 mL culture for 5 min adsorption. Dilutions were performed as described above, and samples were taken every 5 min until the 90-min point. Plaque counts were normalized to the adsorption control, and numbers of PFU per milliliter were calculated and plotted. Nonlinear regression curves were fitted to the data using the sigmoidal model in GraphPad Prism using the least-squares method, and the burst size was calculated by dividing the top plateau average by the bottom plateau average.

### Phage titer assays.

Phage titers were determined as previously described (62). Briefly, recipient strains were grown to an OD_600_ of 0.35, and 100-μL aliquots of recipient were mixed with 100 μL phage lysate at different dilutions in phage buffer (10^0^ to 10^8^; 1 mM MgSO_4_, 4 mM CaCl_2_, 50 mM Tris-HCl [pH 8], 0.1 M NaCl). After 10 min incubation at room temperature, 3 mL of liquid PTA (phage top agar; Oxoid nutrient broth no. 2, agar 3.5% [wt/vol]) was added and the mixture was poured out on phage base (PB) plates (nutrient broth no. 2, agar 7% [wt/vol]). Plates were incubated at 37°C overnight, and plaques were counted. EOP was calculated by dividing the number of PFU per milliliter in the WT by that in the ΔCRISPR mutant and multiplying by 100 to obtain percentages.

### DefenseFinder and PADLOC bioinformatic analyses.

The genome of 110900 (accession no. CP058615.1) was checked for the presence of defense systems using the defense system identification tools PADLOC (52, 53) and DefenseFinder (54), using the standard settings on their respective web servers. For PADLOC, the input file was the NCBI-formatted genome (.gb), while for DefenseFinder, the input file and pipeline were nucleic FASTA, and analysis was preceded by Prodigal.

### Data availability.

Sequencing data are available at doi:10.5281/zenodo.7747092 and via the GitHub repository (https://github.com/KasperMikrobe/S.aureus_CRISPR_strains/tree/CRISPR).
