# Supplementary material for: An Endogenous Staphylococcus aureus CRISPR-Cas System Limits Phage Proliferation and Is Efficiently Excised from the Genome as Part of the SCCmec Cassette
Source: Microbiol Spectr. 2023 Jul 5;11(4):e01277-23. doi: 10.1128/spectrum.01277-23 (PMC10434264; doi:10.1128/spectrum.01277-23)
Supplement: Supplemental file 1 — Supplemental material. Download spectrum.01277-23-s0001.docx, DOCX file, 0.02 MB [file spectrum.01277-23-s0001.docx]

**Supplementary Table 1. Spacer sequences.**

| **Spacer number** | **Sequence 5’-3’** |
| --- | --- |
| 1.1 | TACTTCTCTCGCCATTTCTGCTAATTGTTCTACTTTG |
| 1.2 | TACTAGATACCCAGAACAAAATAGGTCTAACGAAA |
| 1.3 | TTCTATAAGTTCATTAATTCCGATACCTAGATTATCT |
| 1.4 | TTTTTTTCCACCCTTTCAGATCATCTATGATCTTG |
| 1.5 | TAATTTTCTAATTCTATAAGTTCATTAATTCCGAT |
| 1.6 | TTTTTTTCCACCCTTTTCTCTTTATTAAATTATCT |
| 1.7 | TTATACTATTTACATAATTTTTTATGTGTCTGTCTAC |
| 1.8 | TTAATAGTGTTGTTCTCTATTAAAAGATACAATCCTGT |
| 1.9 | TTAGAATGTTATTATCTAAGTGGTCGATGTATTCC |
| 1.10 | TTCATACTAGCACCCCACTCTCTACTGAACAAGTATCA |
| 1.11 | TCTTAAAATCTAATTGCATTGTTATCAATTCCTTTA |
| 1.12 | TTCTGTAATGTATTCATTTAATGTAATCATAATTTTTTC |
| 1.13 | TTAGACCATTTACCTCATTATATTTATAGTCTTTATTA |
| 1.14 | TTTTTCTTTAACTGTTTTTACTGCCCATTTAATAGT |
| 1.15 | TATAAACCCGTTCAATTCGTTATCTTTAAATTCTTG |
| 1.16 | TACAACTTCGTCATCTTTCATCATTTCTCTTACATCA |
| 1.17 | TATATTTCTTCCATGAATAACACCCTCCTTTTTTCTA |
| 1.18 | TAAGTTAACGGCATTACCTAATAAAAATATTTTAGG |
| 1.19 | CTCATCTTTCATGTCACTGATTAATTCATTTGTA |
| 1.20 | CGGTAATAGTTGCTCAATAGGTAATAAAACGTCGGT |
| 2.1 | CTTCTAAGACGCGATATGATTCTAATTGGTCTTC |
| 2.2 | GATATACTCCTTTACCATGTATTAATTCTGGACCACT |
| 2.3 | CATATTCGATCGTGTATATCAAAACTTTATGC |

**Supplementary Table 2. Primer sequences used in study.**

| **Primer** | **Sequence 5’-3’** |
| --- | --- |
| *circR* | CCGCTCCTTTTATATTATGCAC |
| *criF* | GGTTTTTAGCAAAATCACTGATAGG |
| *arsF* | CCGCATCATTAACCGATACG |
| *adsAF* | GCGAAACAACCAGTGCCAAG |
| *adsAR* | CAGCAGTTCCTTCCAATGCG |
| *pIMAY-Z_CRISPR_uF* | CCTCACTAAAGGGAACAAAAGCTGGGTACCGCGCTATCACGTTTAGACCAATC |
| *pIMAY-Z_CRISPR_uR* | GGGATCATCAAGATGTCTAATTTAAAATAAGAGATAAATTTAA |
| *pIMAY-Z_CRISPR_dF* | TTAAATTAGACATCTTGATGATCCCAATTGGCTTG |
| *pIMAY-Z_CRISPR_dR* | CGACTCACTATAGGGCGAATTGGAGCTCGTTGATGGGTAATAACCTAATTTAG |

**Supplementary Figure 1.** The majority of the phiIPLA-RODI lysates that escaped CRISPR targeting in the bioscreen assays contained deletions at the spacer targeting site. The schematic shows the alignment of the variants found following whole genome sequencing of our ancestral WT phiIPLA-RODI lysate and 5 separate samples of phiIPLA-RODI that lysed 110900 WT in the experiment shown in Fig 3. The spacer 1.9 targets the phage at the indicated protospacer sequence in the phage genome. The table below shows the variations in detail, excluding those also present in the sequenced WT phiIPLA-RODI or with an insignificant P-value. “Sample” indicates the phiIPLA-RODI sample number and minimum/maximum are the position in the phage genome.
