# Supplementary figures and images for: An Endogenous Staphylococcus aureus CRISPR-Cas System Limits Phage Proliferation and Is Efficiently Excised from the Genome as Part of the SCCmec Cassette
Source: Microbiol Spectr. 2023 Jul 5;11(4):e01277-23. doi: 10.1128/spectrum.01277-23 (PMC10434264; doi:10.1128/spectrum.01277-23)

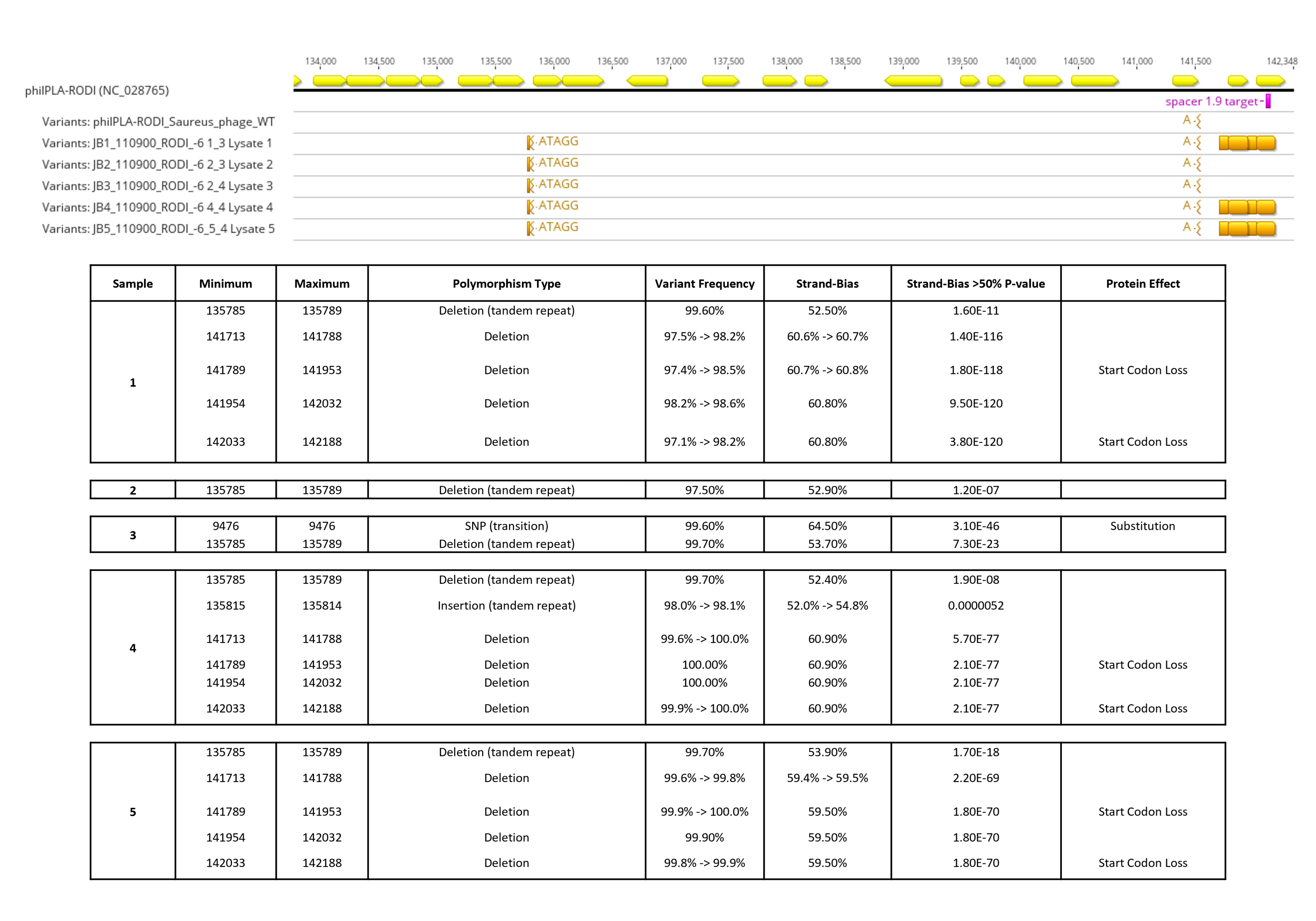

Supplement: Supplemental file 2 — Supplemental material. Download spectrum.01277-23-s0002.png, PNG file, 2.3 MB [file spectrum.01277-23-s0002.png]
